# Caloric Restriction as a Strategy to Improve Vascular Dysfunction in Metabolic Disorders

**DOI:** 10.3390/nu8060370

**Published:** 2016-06-15

**Authors:** Concha F. García-Prieto, María S. Fernández-Alfonso

**Affiliations:** 1 The Rolf Luft Research Center for Diabetes and Endocrinology, Karolinska Institutet, Stockholm 17176, Sweden; concepcion.fernandez.garcia-prieto@ki.se; 2 Instituto Pluridisciplinar and Facultad de Farmacia, Universidad Complutense, Madrid 28040, Spain

**Keywords:** caloric restriction, dietary intervention, endothelial dysfunction, cardiovascular disease, obesity

## Abstract

Caloric restriction (CR) has proved to be the most effective and reproducible dietary intervention to increase healthy lifespan and aging. A reduction in cardiovascular disease (CVD) risk in obese subjects can be already achieved by a moderate and sustainable weight loss. Since pharmacological approaches for body weight reduction have, at present, a poor long-term efficacy, CR is of great interest in the prevention and/or reduction of CVD associated with obesity. Other dietary strategies changing specific macronutrients, such as altering carbohydrates, protein content or diet glycemic index have been also shown to decrease the progression of CVD in obese patients. In this review, we will focus on the positive effects and possible mechanisms of action of these strategies on vascular dysfunction.

## 1. Introduction

Obesity is a chronic disease due to an energetic imbalance in which caloric intake is higher than energetic expenditure. It is closely associated with insulin resistance and type 2 diabetes (T2D), leading to several manifestations of cardiovascular disease (CVD), such as hypertension, coronary artery disease, myocardial infarction, heart failure and stroke [1]. CVD is a major health problem worldwide accounting for 30% of all deaths and demands a global approach to prevention and early detection. In obese individuals, the earliest indication of vascular dysfunction preceding the development of prehypertension and hypertension is the impairment of endothelial function [2]. Therefore, endothelial function improvement should become a key approach to prevent and/or treat cardiovascular (CV) complications related to metabolic disorders. 

The primary goal to reduce CVD risk in subjects who are overweight and obese is a moderate and sustainable weight loss. An improvement of multiple metabolic and hormonal factors implicated in the pathogenesis of CVD associated with metabolic disorders is already achieved by a weight loss of 5%–10% [3] that needs to be maintained over time. Since pharmacological approaches for body weight (BW) reduction have, at present, a poor long-term efficacy, other strategies such as caloric restriction (CR), exercise programs, or bariatric surgery are of great interest [3,4]. 

CR is defined as a state in which energy intake is reduced below usual *ad libitum* intake without malnutrition, independently of its duration. It is one of the most common and cost-effective interventions used to induce BW reduction and CV risk factor amelioration. Other dietary strategies changing specific macronutrients have also been shown to decrease progression of CVD. It is important to note that the induction of a negative energy balance is mandatory for achieving the metabolic benefits of weight loss since the sole reduction in fat mass alone by surgical procedures does not improve CV risk factors in obese patients [5,6]. Taking into consideration the complex and vast literature regarding dietary strategies, in this review, we will focus on the positive effects on vascular dysfunction, CV risk factors and CVD exerted by CR and macronutrients intake modification. 

## 2. CR Reduces CV Risk Factors 

**Figure 1:**
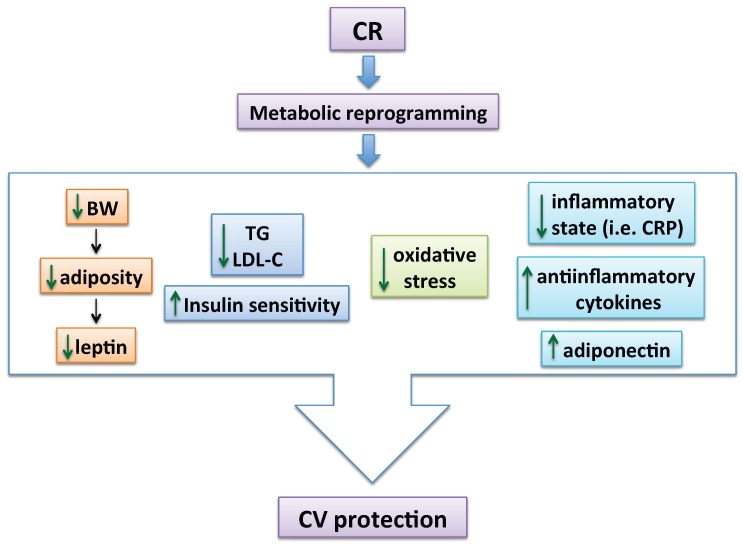
Main mechanisms by which CR exerts CV protection. Reducing the daily caloric intake induces a metabolic reprogramming in both healthy and obese individuals, subsequently leading to CV protection. This includes a reduction in BW and adiposity, thus lessening leptin levels. A decrease in TG and LDL-cholesterol levels together with less oxidative stress and inflammation and an increase in adiponectin levels are some of the main underlying mechanisms described (BW: body weight; CR: caloric restriction; CRP: high-sensitivity C-reactive protein; CV: cardiovascular; LDL-C: low-density lipoprotein -cholesterol; TG: triglycerides).

Benefits on CV risk factors by reducing the daily caloric intake have been widely described in overweight and obese patients [7,8,9,10,11,12,13,14,15,16,17,18,19]. CR induces reductions in BW, waist perimeter, total fat, serum triglycerides (TG), or low-density lipoprotein (LDL)-cholesterol concentrations [12,14,20]. It is also associated with a reduction of circulating insulin levels together with an increase in insulin sensitivity [12,14,18,20]. The decrease in adiposity leads to reductions in leptin [14,18], inflammatory cytokines, prostaglandins, and/or oxidative stress [9,10,14,18], as well as to an increase in the anti-inflammatory Il-10 [14] and adiponectin (Figure 1) (see Section 4.4). In obese patients with or without associated hypertension, weight loss enhances flow-mediated vasodilation (FMD, which determines endothelial function *in vivo*) [8,11], and induces a reduction in blood pressure (BP) [7,20].

Interestingly, benefits of CR are not only observed in obese subjects. Long-term (3–15 years) CR in non-obese humans has a profound beneficial impact on CV risk factors, such as serum total cholesterol, LDL-cholesterol, high-density lipoprotein (HDL)-cholesterol, TG, and BP [20]. A decrease in the inflammatory state is reflected by the extremely low levels of high-sensitivity C-reactive protein (CRP) detected in these subjects [20]. Since CV risk factors increase with age, CR reveals as a promising strategy to prevent the development of CVD in both obese and non-obese individuals. However, it has to be noted that long-term interventions might not be equivalent to short-term interventions in the context of obesity and/or metabolic dysfunction, which may include increased resilience and/or improvements in indices of disease risk but are not associated with delayed aging (Figure 1). 

## 3. CR Protocols Differ in Their Starting Point, Severity, Duration and Number of Phases 

**Figure 2:**
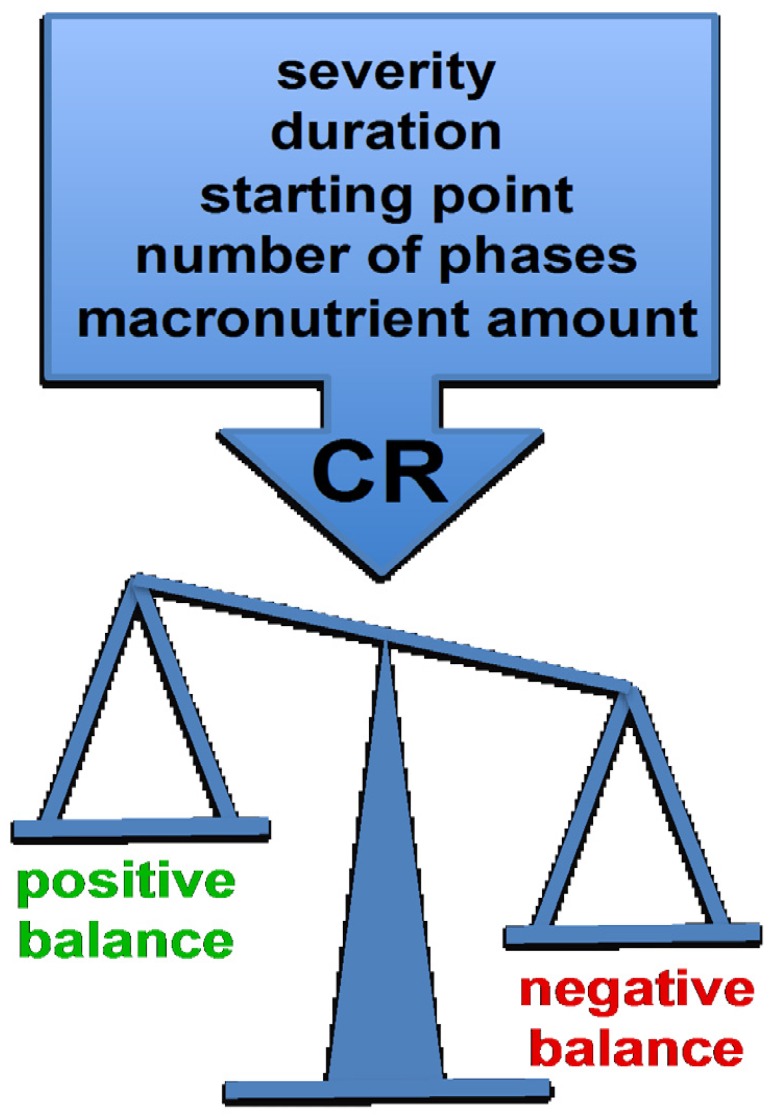
Critical aspects in a CR to achieve the desired effects. Although effects exerted by CR have been widely studied, there is no agreement in how a CR must be in order to prevent CV events. Numerous studies suggest that its severity, duration, starting point, number of phases and composition (*i.e.*, macronutrient amount) are important aspects to take into account. Modifying all these characteristics in different ways can exert a positive or a negative balance, thus affecting CV risk factors (CR: caloric restriction; CV: cardiovascular).

To assess and compare the effectiveness of CR protocols on CVD several aspects, such as CR severity, its duration, starting point and number of phases, need to be taken into account (Figure 2). A comparison of several approaches is shown in Table 1.

There is no agreement on how severe a CR must be in order to confer benefits in different organs and systems. In general, the *ad libitum* (AL) caloric intake is reduced around 20%–40% exerting positive effects without deleterious consequences [21]. However, numerous protocols include an alternate-day fasting, in which caloric intake reduction will be intermittent [22,23]. Most of the CR protocols reduce very intensively the energetic consumption for a long time. Some examples include a daily 30%–40% reduction for 12 months or longer [24,25]. Others use comparable approaches but only for four or five weeks, obtaining similar effects [26]. This suggests that a CR does not need to be prolonged for a long time to be effective, with the advantage that short-term CR is easier to include in clinical practice. In this context, a genomic analysis revealed that the results obtained after CR during four weeks were similar to those from longer CR (28 weeks). In this study, the short-term CR revealed the 70% of the effects observed under the long-term CR on liver gene expression with age [27]. However, other authors report different findings in other tissues, such as in white adipose tissue [28], probably due to variations in responding to fasting cycles. Overall, we feel that most of the studies do not reflect a realistic intervention since they include really severe protocols.

Initially, it was thought that benefits appeared only after long periods of CR [46]. Nevertheless some of the beneficial effects promoted by CR, such as plasmatic glucose levels decrease, show up within the first week of the diet [47]. Important protection of endothelial function occurs in vascular aging models even under CR protocols for less than three weeks [35,48]. Other vascular aged-related complications, such as aortic stiffening and artery wall hypertrophy, need longer dietary treatments to be prevented [29].

Regarding the starting point of a CR, its effects on lifespan are higher if it is initiated at the weaning [49,50,51]. A study in 10-week-old spontaneously hypertensive rats (SHR), with a moderate hypertension, showed that a 10% CR for two weeks followed by a 40% CR for three additional weeks avoided the increase in BP levels [32]. However, starting the same protocol at older ages, when BP values were higher (>200 mmHg) could not reduce those values. This suggests that CR protocols might be more beneficial at early stages of vascular disease [32].

The different phases in which a CR can be subdivided play a substantial role in the achievements can be reached. In multiphase dietary interventions, the pattern of adipokine expression, secretion rate, and plasma levels is different with respect to the phase of the intervention, and to the cellular origin of the respective adipokine [42]. Adipocyte-derived adipokines (adiponectin, leptin, serum amyloid A, or haptoglobin) decrease (except for adiponectin), during the initial very low-calorie diet (VLCD), whereas they increase toward prediet levels during the weight stabilization phase. Similar results have been observed with low-calorie diets [52]. In contrast, expression and secretion rate of stromal-vascular fraction-derived adipokines (tumor necrosis factor α TNF-α, interleukins IL-6, IL-10, IL-8, monocyte chemoattractant protein-1, and plasminogen activator inhibitor-1, PAI-1) increased or remained unchanged during the initial VLCD but decreased during the weight stabilization phase [42]. During the various phases of a dietary weight loss program, adipose tissue macrophages and adipocytes show distinct patterns of gene regulation and association with insulin sensitivity, the regulation of gene expression being dependent on the severity and duration of CR [43].

In conclusion, the optimal CR protocol for each specific situation remains to be determined and standardization will be necessary to allow comparison of the beneficial effects of different dietary approaches on CVD. 

## 4. Mechanisms by Which CR Exerts Vascular Protection in Metabolic Disorders

The improvement of vascular dysfunction associated with metabolic disorders might be due to changes in endothelial function, in the arterial wall structure, or in the paracrine effects of perivascular adipose tissue (PVAT).

### 4.1. Effects of CR on Endothelial Function

CR leads to an improvement of endothelial function in arteries from several models of aged [24,29,33,34,36,53] and obese rodents [38,39,40]. Underlying mechanisms include the increase of nitric oxide (NO) bioavailability [29,31,35,48] due to the enhancement of endothelial nitric oxide synthase (eNOS) expression and/or activity [29,37,54], together with the suppression of vascular oxidative stress associated to superoxide anion (O_2_^−^) production [29,31,34,38,40] and lipid peroxidation [38]. Vasoprotective effects of life-long CR both reduce the expression and activity of NADPH oxidase and O_2_^−^ production, and enhance superoxide dismutase (SOD) activity [29]. 

Endothelial function improvement is also achieved by the reduction of inflammatory mediators, such as CRP, which is a moderately good predictor of acute vascular events related to obesity [55], IL-6 and TNF-α in mice [55], as well as decreases in nuclear factor NF-κB activity [33,34]. 

A key event associated with the improvement of endothelial function under CR is the activation of the AMPK-PI3K-Akt-eNOS signaling pathway. A dysregulation of this pathway in over-nutrition and obesity contributes to the development of endothelial dysfunction (for review, see [56]) in both diet-induced [57] and genetic models of obesity, such as Zucker *fa*/*fa* obese rats [58], Zucker diabetic fatty rats [59] or Otsuka Long Evans Tokushima Fatty rats [60]. CR triggers endothelial AMPK activation leading to (i) normalization of endothelial function and systolic BP reduction in Zucker obese rats [39]; (ii) an improvement of vascular compliance and BP reduction in hypertensive rats [32]; or (iii) revascularization in response to ischemia [26]. Moreover, activation of AMPK by CR reduces lipotoxicity associated with high-fat diets (HFD), insulin resistance, and obesity by decreasing the excessive exposure of the endothelium to free fatty-acids (FFAs) [61,62], thus exerting a protective effect on endothelial cells [61,62]. Obese patients subjected to CR that reported systolic BP reduction, as well as FFA and inflammatory marker level decrease, showed an increase of AMPK phosphorylation in peripheral blood mononuclear cells [19]. The fact that these changes are observed even with a moderate reduction in BW supports the predominant role of the negative energy balance to achieve CV benefits of CR.

Activation of the cellular sensing protein sirtuin-1 (SIRT1) is another mechanism proposed as key mediator of vascular benefits of CR [29,34]. Arterial aging is associated with changes in expression and/or activity of SIRT1 and mammalian target of rapamycin (mTOR), which are ameliorated by life-long CR. In the same line, serum obtained from CR patients activates SIRT1 in human cells *in vitro* [63]. There are, however, very few studies assessing the vascular role of SIRT1 activation by CR in obesity, and further research needs to be performed in this line. 

### 4.2. Effects of CR on Arterial Wall Structure and Remodeling

Subclinical organ damage, such as vascular remodeling, precedes the occurrence of CV events in individuals with obesity and hypertension [64]. Life-long CR significantly reduces large elastic artery wall hypertrophy and prevents aortic stiffening in mice [29] due to a suppression of collagen production [29] and elastin fiber degradation [53]. Increases in elasticity after CR have also been described in arteries from young, but not from aged SHR [32]. This suggests that reversal of early changes in the arterial wall structure, at a time when vascular remodeling is emerging and still reversible, is essential for the prevention of vascular dysfunction.

Mechanisms underlying CR-induced changes in vascular remodeling have been mainly analyzed in models of aging. CR introduced early in life protects against aortic fibrosclerosis by decreasing oxidative damage and consequently reducing the levels of transforming growth factor beta-1 (TGFβ1). This change very likely occurs through a downregulation of the mitogen-activated protein kinases (MAPK), c-Jun N-terminal kinase (JNK) and activator protein-1 (AP-1) signaling pathways [36]. Whether the same mechanisms underlie the effects of CR on metabolic arterial wall remodeling remains to be further analyzed.

Both intervention and cross-sectional studies in humans demonstrate that short-term CR and reduced BW are associated with lower BP and carotid wall thickness [15,16,20,65]. A weight loss of around 8% results in a mean decrease of 0.07 mm in carotid artery diameter, whereas individuals who achieved at least a 5% weight loss showed a significant reduction in mean carotid intima:media thickness [15]. A meta-analysis of 43 studies on moderate weight loss (around 11%) due to CR, with or without exercise, demonstrates an improvement of some arterial compliance and stiffness parameters, such as cardio-ankle vascular and β-stiffness index, arterial compliance and distensibility, distal oscillatory compliance, proximal capacitive compliance, systemic arterial compliance or reflection time. However, other parameters, such as augmentation index, strain, augmentation pressure and pulse pressure were not significantly improved with weight loss [66]. All of these results demonstrate that an intensive dietary intervention at a time when vascular remodeling has only been initiated and is not irreversibly established might significantly reverse some of the key adverse vascular structural changes associated with excess weight in severely obese adults. 

### 4.3. Effects of CR on PVAT Dysfunction in Obesity

Perivascular adipose tissue (PVAT) is the adipose tissue surrounding most blood vessels, except cerebral vessels. It might be white or brown adipose tissue and produces a number of vasoactive factors, adipokines and cytokines. A large body of evidence supports the paracrine influence of PVAT for the maintenance of vascular resistance under physiological and pathophysiological conditions [67]. PVAT releases a number of adipokines, inflammatory cytokines, and other vasoactive factors, which are variable in quantity and pattern depending on the PVAT amount [67]. In fact, obesity triggers an increase in PVAT throughout the vasculature [68,69], accompanied by an unbalance in favor of vasoconstrictor and pro-inflammatory substances, which leads to endothelial dysfunction and vascular damage [70,71,72]. Long-term HFD in mice leads to PVAT dysfunction, characterized by an increase in NADPH oxidase activity and O_2_^−^ release, as well as by a reduction in extracellular SOD expression, total SOD activity, eNOS levels, and NO availability [70]. Similarly, PVAT of New Zealand obese mice show increased O_2_^−^ levels formation and decreased SOD expression, leading to an impaired hydrogen peroxide (H_2_O_2_) production, which contributes to vascular dysfunction reducing the anti-contractile effects of PVAT [73]. In Ossabaw obese swine H_2_O_2_-mediated vasodilatation was markedly attenuated by the presence of coronary PVAT [74]. Ma *et al*. [72] showed that PVAT induces endothelial dysfunction by dysregulation of the AMPK/mTOR pathway in the aorta of diet-induced obese rats, characterized by a downregulation of AMPK-eNOS pathway and a concurrent upregulation of mTOR. Moreover, obese periaortic PVAT high FFA levels could attenuate the anti-contractile properties of PVAT by a TNF-α-dependent [75]. 

An interesting issue, which deserves future investigation, is the impact of the diet composition on oxidative stress in PVAT. A fructose-rich diet, which decreases polyunsaturated FAs and increases saturated and monounsaturated FAs in PVAT impairs vascular function by a decrease in antioxidant enzymes and a reduction in glutathione content [76]. Altogether, these results indicate that obesity and the diet composition induce a switch in PVAT to a more pro-inflammatory, pro-oxidant and vasoconstrictor phenotype.

Since the beneficial or deleterious paracrine influence of PVAT is directly dependent on its amount [77,78], a key question is whether the proportion of adipose tissue loss induced by CR is uniform (overall adiposity) or predominant in specific adipose depots (*i.e.*, PVAT). Significant reduction in the thickness of epicardial fat (surrounding coronary arteries) has been described both in severely obese patients who underwent substantial weight loss after bariatric surgery [79], as well as after a short-term VLCD program [41]. Interestingly, the decrease of epicardial fat is substantially higher than changes in overall BW loss body mass index and waist circumference, and correlates with the improvement in both left ventricular mass and diastolic function. Interestingly, bariatric surgery also reverses the obesity-induced damage to PVAT anticontractile function by reducing adipocyte hypertrophy, PVAT inflammation and increasing both PVAT-derived NO and adiponectin availability [80]. We believe that these studies open a new approach for the management of vascular damage and CV risk associated with PVAT dysfunction in metabolic related disorders. 

### 4.4. Effects of CR on Vascular Actions of Leptin and Adiponectin

Obesity is associated with hyperleptinemia and hypoadiponectinemia, both playing a key role in the pathogenesis of endothelial dysfunction [81]. Chronic hyperleptinemia has been linked to endothelial dysfunction and damage [82,83,84] stimulating the increase in NADPH oxidase activity and O_2_^−^ production in the vascular wall, as well as promoting glutathione peroxidase activation to remove excessive H_2_O_2_ production. On the other hand, hypoadiponectinemia is closely associated with endothelial dysfunction in humans and adiponectin knock-out mice show a decrease in eNOS phosphorilation levels. 

Different CR protocols in rats, markedly changes the adipokine production pattern leading to an increase in circulating levels of adiponectin, whereas leptin levels profoundly decrease in adipose tissue [85,86].

A role for adipokine levels in PVAT and their paracrine influence on the vascular wall might not be discarded. *Ob*/*ob* mice lacking leptin do not exhibit NO production in perivascular adipocytes. Interestingly, this is restored in PVAT after two-week subcutaneous leptin infusion, suggesting that NO release in PVAT seems to be mediated by leptin [87]. Hypoadiponectinemia in patients with T2D stimulates vascular NADPH oxidase expression, which is counterbalanced by upregulation of adiponectin expression in PVAT aimed at suppressing in a paracrine manner NADPH oxidase activity via a PI3K/Akt-mediated deactivation of Rac1 and the downregulation of p22^phox^ gene expression [88]. These studies again stress the concept that PVAT-vessel interaction is a promising therapeutic target for the prevention of vascular complications of metabolic disorders. The effect of CR and dietary approaches on this interaction deserves future investigation.

## 5. Dietary Strategies Based on Macronutrients Modification

Not only the amount but also the quality of the nutrient intake contributes to benefits on vascular function [89,90,91]. However, any dietary change can be universally applied as positive at the CV level since their beneficial effects are different depending on the metabolic state of the individual. Although dietary interventions need to be personalized on the basis of patient requirements, some of the most widely approaches used in clinical practice regarding macronutrients content on the diet and its effects at vascular level are described in Sections 5.1 to 5.3 below.

### 5.1. Low-Carbohydrate Diets

In both overweight and obese patients, carbohydrate restricted diets are more effective than low-fat diets in terms of atherogenic dyslipemia and other metabolic syndrome characteristics such as inflammation, oxidative stress and CV risk markers (*i.e.*, apolipoproteins A-1 and B, LDL particle distribution, and FMD) [92,93,94,95]. Peripheral small artery vascular function is improved with low carbohydrate diets when there is a decrease in BW [96,97]. However, some authors report that the degree of weight loss in obese patients is not correlated to changes in BP, endothelial function and inflammatory markers [98]. Restricting dietary carbohydrate positively impacts lipid homeostasis and inflammatory markers (*i.e.*, TNF-α, IL-6, PAI-1) even when the saturated fat intake is higher due to the isocaloric replacement of carbohydrates [92,93,94,95]. Dysregulation of PAI-1 is involved in enhanced inflammation, vascular damage and subsequent thrombogenicity [99]. Thus, studies with different dietary interventions aim to establish PAI-1 as a possible therapeutic target for controlling CVD in obese patients [44,45]. Moreover, beneficial effects of carbohydrate restriction in combination with pharmacological treatments, *i.e.*, statins, have been described [98]. A decrease in approximately 400 kcal/day for six weeks (with carbohydrates representing the 11% of the daily intake) reduced CV risk markers such as serum TG, soluble E-selectin and intracellular adhesion molecule-1. Both systolic and diastolic BP decreased together with an increase in reactive hyperemia, indicating a better resistance vessel endothelial function, probably in a prostaglandins-mediated way and independent of NO increase [98,100]. 

**Figure 3:**
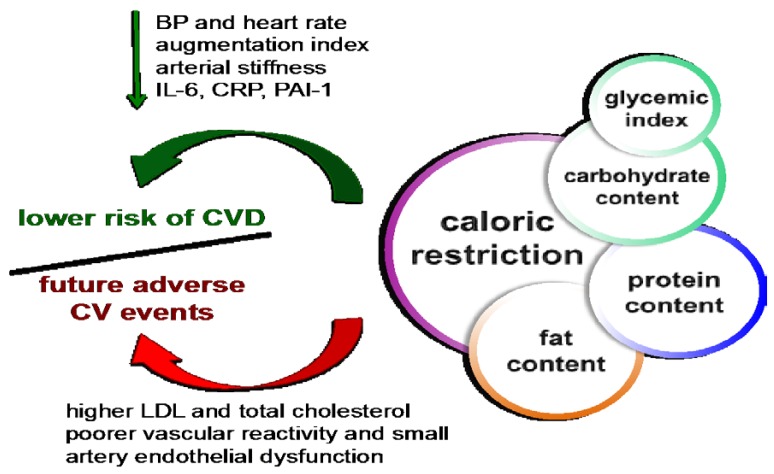
CV outcome of CR depends on macronutrient composition of the diet. Both the amount and the quality of each macronutrient of the diet are important in order to achieve positive effects at CV level. A proper balance in macronutrient amount is key to avoid future adverse CV events (BP: blood pressure; CR: caloric restriction; CRP: high-sensitivity C-reactive protein; CV: cardiovascular; CVD: cardiovascular disease; IL-6: interleukin 6; LDL: low-density lipoprotein; PAI-1: plasminogen activator inhibitor-1).

However, there is no consensus regarding how much the carbohydrate amount of the diet must be reduced to be beneficial. A recent study comparing the effects of two low-carbohydrate diets for 12 weeks in rats (80% energy intake with 34% carbohydrate reduction *vs.* 60% energy intake with 68% carbohydrate reduction of the control diet) demonstrated that decreasing carbohydrate intake to a large extent even with a lower caloric intake has detrimental effects on lipid balance due to an increase in LDL and total cholesterol levels [30]. Indeed, dietary patterns characterized by a low amount of carbohydrate but reciprocally higher content in fat and protein are related to a poorer vascular reactivity in overweight and obese patients [97] and in patients with T2D and increased vascular risk [101]. This emphasizes the necessity of studying the impact of macronutrient composition and nature on CVD (Figure 3).

### 5.2. Higher Protein Content: Does It Make the Difference? 

Diets low in carbohydrates have been proven to reduce BW in both overweight and obese patients [91,96,97,102] and to maintain weight when associated with a moderate increase in protein amount [103]. Low-fat/high-protein diets are more effective for long-term maintenance or further improvement of vascular function [104,105]. Thus, energy-restricted diets are generally compensated by protein content, usually with meal replacements [101,105]. Obese T2D subjects receiving either a meal replacement-based low-calorie diet (approximately 1000 kcal/day) or a low-fat, high-protein, reduced-carbohydrate diet (HP, intake decrease about 600 kcal/day) for eight weeks and either switching to or continuing with the HP diet for 44 additional weeks showed similar positive impacts on endothelial function and inflammatory markers [105]. Both diets reduced blood glucose, TG and LDL-cholesterol and improved endothelial function by lessening soluble E-selectin and increasing FMD. However, only the HP diet decreased CRP and IL-6 levels, markers of inflammation related to vascular homeostasis. Controlled trials in which obese patients were assigned to two periods of a four-week hypocaloric diet (either high in proteins or a conventional one, 1200 kcal/day) [106] showed similar results in some metabolic risk markers after both diets. Interestingly, diets high in protein were associated with improvement in some cardiometabolic risk factors such as PAI-1, vascular endothelial growth factor, fasting plasma glucose and CRP despite the fact of its higher fat content. However, only the conventional hypocaloric diet decreased systolic BP, highlighting the importance of every macronutrient maintaining vascular homeostasis in obese patients [106]. Since high CRP precedes atheromatic events [107], lessening its levels could pave the way to better prevent CV disease. Some clinical trials (*i.e.*, the OmniHeart study) support the idea that a partial replacement of carbohydrates with either protein or monounsaturated fat has additional beneficial effects on the basis of a healthy diet at a vascular level independent of BW loss in both non-obese and obese patients with prehypertension or stage 1 hypertension [108]. Without reducing caloric intake (2100 kcal/day), these patients further reduced systolic and diastolic BP and decreased estimated risk of suffering coronary heart disease with both diets rich in protein or rich in unsaturated fat [108].

Besides the positive effects that a protein supplementation can exert, replacing carbohydrates with higher amounts of protein and fat lead to a low small artery reactive hyperaemia index, a marker of small artery endothelial function [101], which correlates with endothelial dysfunction [109], thus predicting future adverse CV events [110]. Hence, balancing the amount of each macronutrient of the diet might be of importance for achieving positive effects at the CV level (Figure 3).

### 5.3. Diet Glycemic Index Variation

Both the carbohydrate and fat content of a diet influences the occurrence of diabetes and CVD [102,111]. Although diets restricting carbohydrate consumption are a well-known strategy for weight loss with the subsequent improvement of metabolic-related complications in overweight and obese patients [102], recent studies show that following these diets for a long time does not always protect from CVD [101,112,113]. In this context, changing carbohydrate quality rather than quantity has emerged as a better approach to dealing with CV risk factors. Glycemic index (GI) is the quantification of the blood glucose response to a carbohydrate compared to a carbohydrate reference [114]. That is, foods with similar carbohydrate content can behave differently in terms of raising blood glucose. Thus, varying GI might be useful in the management of these diseases (Figure 3). The effects on metabolism exerted by different GI diets are diverse [89,91,115]. Control trials in which obese patients at increased CV risk were subjected to hypocaloric diets with different GI for three months showed a decrease in BW, plasma glucose, total cholesterol, LDL-cholesterol, TG, systolic BP and heart rate independent of the GI of the diet [91]. Interestingly, FMD was influenced, being higher after following the low GI diet [91], a fact observed in studies assessing the acute effects of different GI diets [89]. In this context, the EVIDENT study (2010, Spain) aims to establish an association between GI and vascular homeostasis by assessing arterial stiffness (via pulse wave velocity measurement) and augmentation index (vascular aging related to endothelial dysfunction) [116]. Their latest results show that an increase in GI correlates with an enhancement of augmentation index, thus increasing the risk of suffering a CVD [116]. Low GI diets can reduce oxidative stress [117] and inflammation [90], factors widely associated with endothelial dysfunction. In this context, low GI hypocaloric diets have been shown to decrease CRP blood levels in overweight patients [90]. However, some clinical trials claim that GI is not related to an improvement in CV risk factors on the basis of a healthy diet [118]. In this controlled feeding study, the effects of four different interventions on carbohydrate content from a healthy diet without losing weight were analyzed. No differences were observed between high and low GI diets regarding insulin sensitivity, lipid homeostasis and systolic BP [118]. All of these diverse results between studies may be due to changes in content of total carbohydrates and fibers, no concomitant weight loss and duration of the dietary treatment, although long-term studies with BW decrease conclude in a similar way [90,119].

## 6. Conclusions 

All the described dietary modifications are effective by mitigating CV risk factors in obesity. However, despite all the efforts made to establish CR or a balanced diet as the main strategies to either prevent or treat CV complications related to metabolic disorders, there is no fully effective formula, and factors such as severity, duration, starting point, number of phases and composition need to be taken into account. It is important to note that an intensive dietary intervention at a time when vascular dysfunction has only been initiated and is not irreversibly established might significantly reverse some of the key adverse vascular changes associated with obesity. 

On the other hand, many mechanisms underlying CR-induced changes in vascular dysfunction have been mainly analyzed in models of aging (*i.e.*, vascular remodeling). Whether the same mechanisms underlie the effects of CR on obesity related vascular dysfunction remains to be further analyzed. In addition, most of these regimens have the ability to attenuate some, but not all, of the components involved in this complicated multifactorial condition. Therefore, further research is needed to understand in depth the mechanisms involved in the positive effects of CR and macronutrient intake modification on vascular dysfunction associated with being overweight and obese. 

Moreover, we need to define specific biomarkers to (i) assess and/or predict the effect CR on CV risk and (ii) design an optimal and more personalized dietary regime, taking into account individual differences in age, metabolic function and CV risk factors.

## Figures and Tables

**Table 1:** Comparison of several caloric restriction (CR) protocols in rodents and humans.

Reference	Model	CR Protocol
Kondo *et al.*, 2009 [26]	C57/BL6 mice	35% CR for 4 weeks
Donato *et al.*, 2013 [29]	mice	10% CR (1 week) + 25% CR (1 week) + 40% CR throughout the life of the animal
Chen *et al.*, 2015 [30]	Wistar rats	20% CR or 40% CR for 12 weeks—only reduction of starch
Chou *et al.*, 2010 [31]	Wistar rats	40% CR for 2 weeks
Dolinsky *et al.*, 2010 [32]	Wistar and SHR rats	10% CR (2 weeks) + 40% CR (3 weeks)
Chandrasekar *et al.*, 2001 [33]	Fisher344 rats	40% CR for 10 months
Csiszar *et al.*, 2009 [34]	Fisher344 rats	40% CR (life-long; age-related studies)
Ahmet *et al.*, 2011 [24]	Fisher344 rats	40% CR for 22 months (age-related studies)
Zanetti *et al.*, 2010 [35]	Fisher344 rats	26% CR for 3 weeks (age-related studies)
Castello *et al.*, 2005 [36]	Sprague Dawley rats	40% CR for 4, 10 or 22 months (age-related studies)
Ozbek *et al.*, 2013 [37]	Sprague Dawley rats	40% CR for 3 months
Minamiyama *et al.*, 2007 [38]	type II diabetic rats (OLETF)	30% CR for 13 weeks
García-Prieto *et al.*, 2015 [39]	Zucker obese rats	20% CR for 2 weeks
Ketonen *et al.*, 2010 [40]	C57Bl/6J mice under HFD	30% CR for 50 days (with HFD)
Iacobellis *et al.*, 2008 [41]	patients	VLCD (900 kcal/day). Phase 1—complete meal replacement (12 weeks); phase 2—transition period including healthy foods and partial meal replacement (4–6 weeks); phase 3—long-term maintenance
Kitada *et al.*, 2013 [19]	overweight patients	25% CR for 7 weeks
Siklova-Vitkova *et al.*, 2012 [42]	obese patients	800 kcal/day (1 month) + weight stabilization period (low-calorie diet for 2 months + weight maintenance diet for 3 months)
Capel *et al*, 2009 [43]	obese patients	800 kcal/day (1 month) + weight stabilization period (low-calorie diet for 2 months + weight maintenance diet for 3–4 months)
Davì *et al.*, 2002 [9]	obese patients	1200 kcal/day for 12 weeks
Ziccardi *et al.*, 2002 [10]	obese patients	1300 kcal/day for 12 months
Raitakari *et al.*, 2004 [11]	obese patients	580 kcal/day for 6 weeks
Cooper *et al.*, 2012 [15]	obese patients	CR to produce a 8%–10% weight loss within 12 months with or without physical activity
Morel *et al.*, 2011 [44]	obese patients	600 kcal/day (1 month) + 1200 kcal/day (1 month)
Fontana *et al.*, 2007 [13]	overweight/obese patients	16% CR (3 months) + 20% CR (9 months)
Ho *et al.*, 2015 [18]	overweight/obese patients	CR to produce a 5%–7% weight loss within 12 months
Murakami *et al.*, 2007 [45]	overweight/obese patients	≈1200 kcal/day (women) or 1600 kcal/day (men) for 12 weeks with or without exercise program
Sasaki *et al.*, 2002 [8]	obese patients with hypertension	800 kcal/day for 2 weeks

AL: *ad libitum;* CR: caloric restriction; HFD: high-fat diet; SHR: spontaneously hypertensive rats; VLCD: very low-calorie diet.

## Abbreviations

The following abbreviations are used in this manuscript:

Akt	protein kinase B
AL	*ad libitum*
AMPK	adenosine monophosphate-activated protein kinase
AP-1	activator protein-1
BP	blood pressure
BW	body weight
CR	caloric restriction
CRP	high-sensitivity C-reactive protein
CV	cardiovascular
CVD	cardiovascular disease
eNOS	endothelial nitric oxide synthase
FA	fatty acid
FFAs	free fatty-acids
FMD	flow-mediated dilation
GI	glycemic index
H_2_O_2_	hydrogen peroxide
HDL	high-density lipoprotein
HFD	high-fat diet
HP	low-fat, high-protein, reduced-carbohydrate
IL	interleukin
JNK	c-Jun N-terminal kinase
LDL	low-density lipoprotein
MAPK	mitogen-activated protein kinases
MCP-1	monocyte chemoattractant protein-1
mTOR	mammalian target of rapamycin
NF-κB	nuclear factor κB
NO	nitric oxide
O_2_^−^	superoxide anion
PAI-1	plasminogen activator inhibitor-1
PI3K	phosphoinositide 3-kinase
PVAT	perivascular adipose tissue
SHR	spontaneously hypertensive rats
SIRT1	sirtuin-1
SOD	superoxide dismutase
T2D	type 2 diabetes
TG	triglycerides
TGFβ1	transforming growth factor beta-1
TNF-α	tumor necrosis factor α
VLCD	very low-calorie diet
